# Isoprenylated Flavonoids as Ca_v_3.1 Low Voltage-Gated Ca^2+^ Channel Inhibitors from *Salvia digitaloides*

**DOI:** 10.1007/s13659-021-00307-y

**Published:** 2021-04-24

**Authors:** Jian-Jun Zhao, Song-Yu Li, Fan Xia, Ya-Li Hu, Yin Nian, Gang Xu

**Affiliations:** 1 State Key Laboratory of Phytochemistry and Plant Resources in West China, Kunming Institute of Botany, Chinese Academy of Sciences, and Yunnan Key Laboratory of Natural Medicinal Chemistry, Kunming, 650201 People’s Republic of China; 2 University of Chinese Academy of Sciences, Beijing, 100049 People’s Republic of China

**Keywords:** *Salvia digitaloides*, Isoprenylated flavonoid, Ca_v_3.1 low voltage-gated Ca^2+^ channel (LVGCC)

## Abstract

Saldigones A–C (**1**, **3**, **4**), three new isoprenylated flavonoids with diverse flavanone, pterocarpan, and isoflavanone architectures, were characterized from the roots of *Salvia digitaloides*, together with a known isoprenylated flavanone (**2**). Notably, it’s the first report of isoprenylated flavonoids from *Salvia* species. The structures of these isolates were elucidated by extensive spectroscopic analysis. All of the compounds were evaluated for their activities on Ca_v_3.1 low voltage-gated Ca^2+^ channel (LVGCC), of which **2** strongly and dose-dependently inhibited Ca_v_3.1 peak current.

**Supplementary Information:**

The online version contains supplementary material available at 10.1007/s13659-021-00307-y.

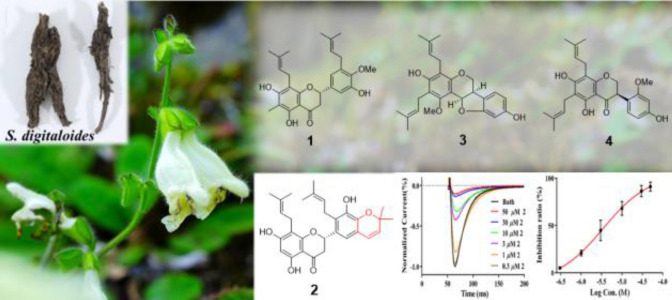

## Introduction

*Salvia*, one of the largest genera of the widespread family Lamiaceae, is distributed widely in the temperate and warm zones worldwide [1]. The name *Salvia* was derived from the Latin word “Salvare”, meaning “to heal or to be safe and unharmed”, which rose the folkloric belief of its “magical” remedial function for various indispositions [2, 3]. Phytochemical studies on more than 130 *Salvia* species have led to an array of secondary metabolites, such as diterpenoids, phenolic acids, triterpenoids, sesquiterpenoids, sesterterpenoids, steroids, as well as flavonoids [1–7]. Diterpenoids with abietane or clerodane skeletons are considered as the main bioactive and characteristic chemical constituents of this genus [4, 5, 7]. Many diterpenoids from *Salvia* plants have shown antimicrobial, antioxidant, anti-HIV, antibacterial, antineoplastic, antiplatelet aggregation activities [3–8]. Attracted by their diverse chemical structures and extensive biological activities, many scientists have engaged in the phytochemistry, pharmacy, chemical synthesis, and biosynthesis of the chemical constituents of *Salvia* plants [2, 3, 5, 6].

**Fig. 1:**
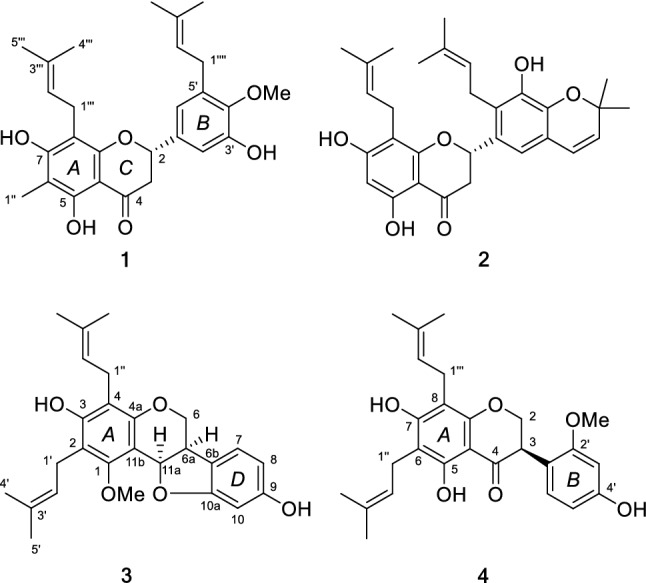
Structures of compounds **1**–**4**

Our group has been committed into the phytochemical studies of genus *Salvia* since 2000, and discovered a series of novel terpenoids with diverse structures and bioactivities [9–14]. As a part of our ongoing research about the plants of *Salvia* species from southwestern China, unexpectedly, three new isoprenylated flavonoids saldigones A–C (**1**, **3**, **4**) (Fig. 1) with various flavanone, pterocarpan, and isoflavanone architectures, were isolated from the roots of *S. digitaloides*, along with a known isoprenylated flavonone, 5,7,3′-trihydroxy-4′,5′-(2⁗,2⁗-dimethylpyran)-8,2′-di(3-methyl-2-butenyl)-(2S)-flavanone (**2**), obtained from *Dendrolobium Ianceolatum* [15]. Terpenoids (accounted for more than 80%) and phenolic acid are the main chemical constituents of the *Salvia* species, while isoprenylated flavonoids from *Salvia* were reported for the first time [3–6]. For the two characterized isoprenylated flavanones (**1** and **2**), compound **1** owns both methylation and isoprenylation substitutions, and **2** has three isoprenyl groups in which one was further cyclized to form a 2,2-dimethylpyran unit. Moreover, **3** and** 4** were characterized as a *cis* configurated pterocarpan and an isoflavone skeletons with two isoprenyl substituents, respectively.

Previous investigation indicated that some isoprenylated flavonoids led to an enhancement of activities or properties compared to the nonprenylated ones, such as cytotoxicity and bacterial neuraminidase inhibitory activities, but no low voltage-gated Ca^2+^ channel (LVGCC) activators have been identified [15–18]. Interestingly, isoprenylated substituents also appear to be important for anchoring certain natural products to the LVGCC [19]. In this study, the biological studies revealed that compound **2** can strongly and dose-dependently inhibited the peak currents of Ca_v_3.1 low voltage-gated Ca^2+^ channel (LVGCC), an important therapeutic target for absence epilepsy, neuropathic pain, Parkinson’s disease, and insomnia [20–22], with an IC_50_ value of 3.5 *μ*M. Meanwhile, **1**, **3**, and **4** were inactive at the concentration of 30 *μ*M. In this note, the isolation and structural elucidation of these compounds were described, as well as their inhibitions on Ca_v_3.1 LVGCC.

## Result and Discussion

Saldigone A (**1**) was isolated as colorless gum and its molecular formula was determined as C_27_H_32_O_6_ by HRESIMS (*m/z* 451.2128 [M – H]^−^, calcd C_27_H_31_O_6_, 451.2126). The IR spectrum displayed bands for hydroxy (3411 cm^−1^) and carbonyl (1626 cm^−1^). The presence of a flavanone skeleton was evident from the characteristic absorption bands at λ_max_ 345 and 297 nm in the UV spectra, the ABX-type spin system (*δ*_H_ 5.26, 1H, dd, *J* = 12.8, 3.0 Hz, H-2; 2.78, 1H, dd, *J* = 17.0, 3.0 Hz, H-3*α*; and 2.97, 1H, dd, *J* = 17.0, 12.8 Hz, H-3*β*) in the ^1^H NMR spectrum (Table 1), conjugated with the oxygenated carbon resonance at *δ*_C_ 78.8 (C-2), a methylene resonance at *δ*_C_ 43.6 (C-3), and a carbonyl resonance at *δ*_C_ 196.7 (C-4) in the ^13^C NMR spectra [23–25]. The ^13^C NMR and DEPT data presented a total of 27 carbon signals attributable to three signals for C-2, C-3, and C-4 mentioned above, 12 signals for two phenyls, as well as 12 other signals for one methyl (*δ*_C_ 7.1, C-1″), one methoxyl group (*δ*_C_ 61.5, MeO-4′), and two isoprenyls (*δ*_C_ 22.2, C-1‴; 121.9, C-2‴; 133.6, C-3‴; 26.0, C-4‴; 18.1, C-5‴/28.4, C-1⁗; 122.2, C-2⁗; 136.0, C-3⁗; 26.0, C-4⁗; 18.1, C-5⁗). Except for the signals for H-2 and H-3, three hydroxy groups (*δ*_H_ 12.25, 1H, s, OH-5; 6.14, 1H, s, OH-7; 5.64, 1H, s, OH-5′) and one methoxy group (*δ*_H_ 3.79, 3H, s, OMe-4′) can also be easily distinguished in the ^1^H NMR spectrum.

**Table 1:** ^13^C (150 MHz) and ^1^H (600 MHz) NMR data of **1** (in CDCl_3_)

No.	*δ*_C_, type	*δ*_H_, mult. (*J* in Hz)	No.	*δ*_C_, type	*δ*_H_, mult. (*J* in Hz)
2	78.8, CH	5.26, dd (3.0, 12.8)	1″	7.1, CH_3_	2.02, s
3	43.6, CH_2_	2.78, dd (17.0, 3.0)	1‴	22.2, CH_2_	3.35, t (6.4)
		2.97, dd (17.0, 12.8)	2‴	121.9, CH	5.20, br t
4	196.7, C		3‴	133.6, C	
5	160.1, C		4‴	26.0, CH_3_	1.73, s
6	104.7, C		5‴	18.1, CH_3_	1.71, s
7	162.2, C		1⁗	28.4, CH_2_	3.35, t (6.4)
8	105.3, C		2⁗	122.2, CH	5.24, overlap
9	157.3, C		3⁗	136.0, C	
10	103.0, C		4⁗	26.0, CH_3_	1.73, s
1′	135.8, C		5⁗	18.1, CH_3_	1.76, s
2′	111.2, CH	6.91, d (2.0)			
3′	149.3 C		OMe-4′	61.5, CH_3_	3.79, s
4′	145.3, C		OH-5		12.25, s
5′	135.3, C		OH-7		6.14. s
6′	119.1, CH	6.73, d (2.0)	OH-3′		5.64. s

**Fig. 2:**
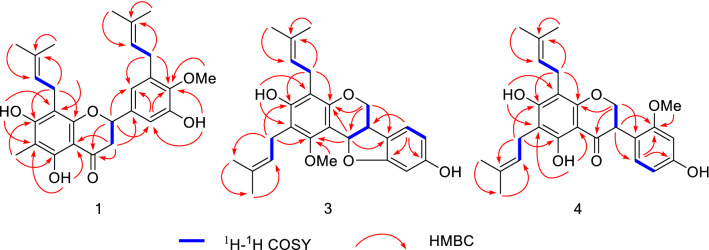
Key HMBC and ^1^H-^1^H COSY correlations of **1**, **3**, **4**

The locations of these oxygenated substituents as well as the isoprenyl and methyl groups were determined by the HSQC and HMBC experiments (Fig. 2). The cross-peaks of OH-5 with C-5 (*δ*_C_ 160.1), C-6 (*δ*_C_ 104.7), and C-10 (*δ*_C_ 103.0), and the methyl proton (*δ*_H_ 2.02, 3H, s, H-1″) with C-5, C-6, and C-7 (*δ*_C_ 162.2) indicated that the hydroxyl group was attached to C-5 while the methyl was substituted at C-6. The correlations of OH-7 with C-6, C-7, and C-8 (*δ*_C_ 105.3), and a set of isoprenyl protons (*δ*_H_ 3.35, 2H, t, H-1‴) with C-7, C-8, and C-9 (*δ*_C_ 157.3) indicated that the isoprenyl group located at C-8 while the hydroxyl group was attached to C-7 of A-ring. In addition, B ring was established as a 3′-hydroxy-4′-methoxy-5′-isoprenyl substitutions by the HMBC correlations of OH-3′ with C-2′ (*δ*_C_ 111.2), C-3′ (*δ*_C_ 149.3), and C-4′ (*δ*_C_ 145.3), of the methoxyl with C-4′, and of the isoprenyl protons (*δ*_H_ 3.35, 2H, t, H-1⁗) with C-2′, C-3′, and C-4′. The ^1^H-^1^H COSY correlation between H-2 and H-3, and HMBC correlations of H-2 with C-4, C-9, C-2′, and C-6′ (*δ*_C_ 119.1), and of H-3 with C-4, C-10 (*δ*_C_ 103.0) and C-1′ (*δ*_C_ 135.8) confirmed the existence of C ring furthermore.

**Fig. 3:**
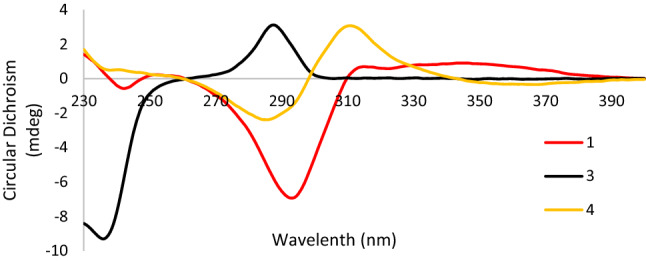
CD spectra of compounds **1** (red), **3** (black) and **4** (yellow)

Biogenetically, flavanones are formed by cyclization of chalcones mediated by the enzyme chalcone isomerase (CHI) in a highly stereospecific manner through a Michael nucleophilic reaction involving the 2′-OH group and the *α*, *β*-unsaturated ketone of the chalcone, leading almost exclusively to the (2*S*)-isomer [26, 27]. As for compound **1**, the circular dichroism (CD) spectra gave the sequential positive and negative Cotton effects near 330 and 280 nm for the *n*-π* and *π*-π* electronic transitions (Fig. 3), respectively, which were consistent with the absolute configuration 2*S* according to the reported data [25, 28–31]. Therefore, the structure of **1** was determined as (2*S*)-5,7,3′-trihydroxy-6-methy-8,5′-diisoprenyl-4′-methoxy flavanone named saldigone A.

The structure of isoprenylated flavonone **2** was elucidated by analysis of 1D, 2D NMR, and CD spectral data confirmed by literature data comparison [15].

Saldigone B (**3**) was isolated as a white powder and was assigned the molecular formula C_26_H_30_O_5_, based on HRESIMS (*m*/*z* 421.2021 [M – H]^−^, calcd. C_26_H_29_O_5_, 421.2020). The IR spectrum displayed bands for hydroxy (3427 cm^−1^). The UV spectrum showed absorption bands at λ_max_ 286 nm. In the ^1^H NMR spectrum (Table 2), the characteristic set of signals at *δ*_H_ 3.35 (1H, br t, *J* = 6.6 Hz, H-6a), *δ*_H_ 4.19 (1H, dd, *J* = 11.0, 4.9 Hz, H-6*α*), *δ*_H_ 3.57(1H, br t, *J* = 11.0 Hz, H-6*β*), and *δ*_H_ 5.65 (1H, d, *J* = 6.6 Hz, H-11a), together with the corresponding carbon signals at *δ*_C_ 39.2, *δ*_C_ 66.7, *δ*_C_ 76.7 for the typical C_3_ unit (C-6a, C-6, and C-11a) in the ^13^C NMR displayed that **3** possessed a pterocarpan skeleton [18, 32, 33]. Its ^13^C and DEPT NMR spectra displayed 26 carbon resonances attributable to the C_3_ unit mentioned above, 12 signals for two phenyls, as well as 11 other signals for one methoxyl (*δ*_C_ 63.2, MeO-1), and two isoprenyls groups (*δ*_C_ 23.4, C-1′; 122.7, C-2′; 134.8, C-3′; 18.2, C-4′; 18.1, C-5′/22.5, C-1″; 122.5, C-2″; 133.6, C-3″; 26.0, C-4″; 26.0, C-5″). The pterocarpan framework can be further evidenced by the ^1^H-^1^H COSY cross peaks of H-6/H-6a/H-11a, couple with the HMBC correlations from H-6 to C-4a (*δ*_C_ 152.9), C-11a, and C-6b (*δ*_C_ 119.8) as well as from H-11a to C-1 (*δ*_C_ 157.1), C-4a, C-11b (*δ*_C_ 106.7), and C-6b (Fig. 2).

**Table 2:** ^13^C (150 MHz) and ^1^H (600 MHz) NMR data of **3** (in CDCl_3_)

No.	*δ*_C_, type	*δ*_H_, mult. (*J* in Hz)	No.	*δ*_C_, type	*δ*_H_, mult. (*J* in Hz)
1	157.3, C		11a	76.7, CH	5.65, d (6.6)
2	114.2, C		11b	106.7, C	
3	155.6, C		1′	23.4, CH_2_	3.37, br t
4	112.1, C		2′	122.7, CH	5.22, t (6.4)
4a	152.9, C		3′	134.8, C	
6	66.7, CH_2_	4.19, dd (4.9, 11.0)	4′	18.2, CH_3_	1.75, s
		3.57, br t, (11.0)	5′	18.1, CH_3_	1.81, s
6a	39.2, CH	3.35, br t, (6.6)	1″	22.5, CH_2_	3.31, d (6.4)
6b	119.8, C		2″	122.5, CH	5.17, t (6.4)
7	125.0, CH	7.05, d (7.7)	3″	133.6, C	
8	107.5, CH	6.33, d (7.7)	4″	26.0, CH_3_	1.68, s
9	157.1, C		5″	26.0, CH_3_	1.73, s
10	98.7, CH	6.37, s	OMe-1	63.2, CH_3_	3.88, s
10a	161.2, C		OH-3		5.66, s

According to the molecular formula (C_26_H_30_O_5_), there should be two OH groups in the structure except for the signals for the two isoprenyls, one methoxy, and 9 signals (including H-6, 2H; 6a, 1H; H-7, 1H, d, *J* = 7.7 Hz; H-8, 1H, d, *J* = 6.33; H-10, 1H, s; H-11a, 1H, and two oxygen atoms) for the pterocarpan skeleton. One of the two OH groups were located on C-3 (*δ*_C_ 155.6) by the HMBC correlations of OH-3 (*δ*_H_ 5.66, s) with C-2 (*δ*_C_ 114.2), C-3, and C-4 (*δ*_C_ 112.1). Then, the remaining OH group was attached on C-9 evidenced by the side-by-side comparison of the NMR spectral data with those of eryvarin J [32], as well as the HMBC correlations of H-7/8/10 with distinguished oxygenated quaternary carbon signal for C-9 at *δ*_C_ 157.1.

Structurally, there are two chiral centers (C-6a, 11a) in the pterocarpans skeleton whose absolute configurations are exclusively *cis* (6a*R*/11a*R* or 6a*S*/11a*S*) over the *trans* in nature [23, 28]. As reviewed previously, the positive ^1^L_b_-band (260–310 nm) and negative ^1^L_a_-band (220–240 nm) indicated the (6a*R*, 11a*R*)-*cis* configuration of pterocaroans [22, 28]. As for compound **3**, the diagnostic positive Cotton effect at 287 nm (Δ*ε* + 8.35) and negative Cotton effect at 236 nm (Δ*ε* − 25.07) observed in the CD spectrum (Fig. 3) conformed the *cis* configuration of **3** as well as both the *R* absolute configurations of C-6a and C-11a [19, 22, 23]. Then, **3** was characterized as (6a*R*,11a*R*)-3,9-dihydroxy-2,4-diisoprenyl-1-methoxy pterocarpan named saldigone B.

Saldigone C (**4**) was isolated as pale-yellow gum and assigned a molecular formula of C_26_H_30_O_6_, based on HRESIMS (*m*/*z* 437.1968 [M – H]^−^, calcd. C_26_H_29_O_6_, 437.1970). The IR spectrum displayed bands for hydroxy (3429 cm^−1^) and carbonyl (1618 cm^−1^). It was deduced to be an isoflavanone based on the characteristic absorption bands in the UV spectrum (λ_max_ 254 and 296 nm) together with three typical aliphatic proton signals (*δ*_H_ 4.47, 1H, br t, *J* = 11.1 Hz, H-2*α*; 4.41, 1H, dd, *J* = 11.1, 5.5 Hz, H-2*β*; 4.27, 1H, dd, *J* = 11.1, 5.5 Hz, H-3) in the ^1^H NMR spectrum (Table 3). This deduction can be further confirmed by the corresponding oxygenated methylene resonance (*δ*_C_ 70.7, C-2), a methine resonance (*δ*_C_ 46.9, C-3), and a carbonyl resonance at (*δ*_C_ 198.2, C-4) exclusively for an isoflavanone skeleton observed in the ^13^C NMR spectra [34, 35]. Furthermore, the ^13^C NMR and DEPT data displayed that the presence of totally 26 carbon signals owing to the signals for C-2, C-3, and C-4 described above, 12 signals for two phenyls, as well as 11 other signals for one methoxyl (*δ*_C_ 55.8, MeO-2′) and two isoprenyls (*δ*_C_ 21.5, C-1″; 122.1, C-2″; 134.9, C-3″; 18.1, C-4″; 26.1, C-5″/21.9, C-1‴; 122.4, C-2‴; 134.2, C-3‴; 18.1, C-4‴; 26.1, C-5‴).

**Table 3:** ^13^C (150 MHz) and ^1^H (600 MHz) NMR data of **4** (in CDCl_3_)

No.	*δ*_C_, type	*δ*_H_, mult. (*J* in Hz)	No.	*δ*_C_, type	*δ*_H_, mult. (*J* in Hz)
2	70.7, CH_2_	4.47, br t (11.1)	6′	131.1, CH	6.92, d (8.3)
		4.41, dd (11.1, 5.5)	1″	21.5, CH_2_	3.33, d (7.1)
3	46.9, CH	4.27, dd (11.1, 5.5)	2″	122.1, CH	5.23, t (7.1)
4	198.2, C		3″	134.9, C	
5	159.9, C		4″	18.1, CH_3_	1.71, s
6	107.3, C		5″	26.1, CH_3_	1.76, s
7	162.2, C		1‴	21.9, CH_2_	3.27, d (7.1)
8	106.3, C		2‴	122.4, CH	5.18, t (7.1)
9	158.4, C		3‴	134.2, C	
10	103.4, C		4‴	18.1, CH_3_	1.73, s
1′	115.6, C		5‴	26.1, CH_3_	1.79, s
2′	158.8, C		OMe-2′	55.8, CH_3_	3.74, s
3′	99.8, CH	6.43, d (2.0)	OH-5		12.53, s
4′	156.8, C		OH-7		6.31, s
5′	107.6, CH	6.36, dd (2.0, 8.3)			

Side-by-side comparison of the 1D NMR data of **4** with those of eryzerin B indicated their structures were closely similar to each other except for the existence for an additional hydroxyl group (*δ*_H_ 12.53) in **4** as evidenced by the MS and 1D NMR [35]. And the location of this OH group on C-5 was deduced by its HMBC correlations with C-5 (*δ*_C_ 159.9), C-6 (*δ*_C_ 107.3), and C-10 (*δ*_C_ 103.4). In addition, the HMBC correlations of the isoprenyl protons at *δ*_H_ 3.33 (1H, d, *J* = 7.1 Hz, H-1″) and *δ*_H_ 3.27 (1H, d, *J* = 7.1 Hz, 1‴) with C-5/6/7(*δ*_C_ 162.2) and C-7/8 (*δ*_C_ 106.3)/9 (*δ*_C_ 158.4), respectively, as well as of methoxy protons (*δ*_H_ 3.74, 3H, s, MeO-2′) to C-2′ (*δ*_C_ 158.8) indicated that the isoprenyls and one methoxyl groups were attached to C-6, C-8, and C-2′, respectively.

To analogy the data for similarly substituted isoflavanone systems, the positive Cotton effect near 310 nm and the negative Cotton effect near 280 nm (Fig. 3) indicated that the absolute configuration at C-3 was *R* for **4** [25, 28, 35]. Thus, **4** was characterized as (3*R*)-5,7,4′-thihydroxy-2′-methoxy-6,8-diisoprenyl isoflavanone named saldigone C.

**Fig. 4:**
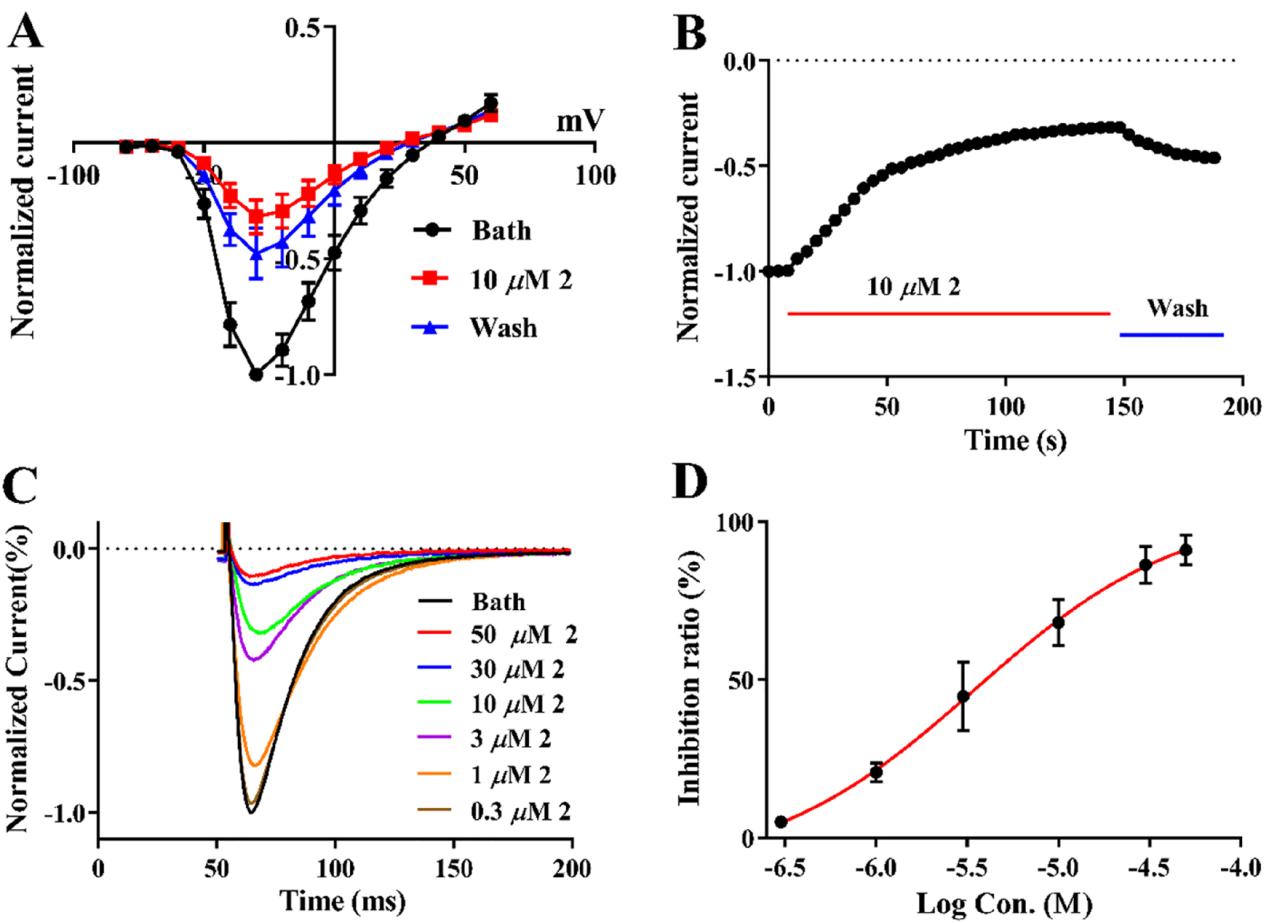
Inhibitory effect of Compound** 2** on Ca_v_3.1. **A** Normalized current–voltage (I–V) curves of Ca_v_3.1 in the absence or presence of 10 *μ*M **2**. Ca_v_3.1 Currents were evoked from a holding potential (HP) of − 100 mV by 150 ms depolarization from − 80 to + 60 mV in 10 mV increment at 4 s intervals. Data are represented as mean ± SD (n = 3). **B** Time course of peak current inhibition by 10 *μ*M **2**. Ordinate axis, peak current during exposure to **2**, which normalized by the peak current after drug exposure. **C** Representative Ca_v_3.1 peak current trace that elicited by 150 ms depolarization to − 40 mV at 4 s intervals from a HP of − 100 mV in the absence (Bath) and presence of various concentrations of **2**. **D** Dose–response curve of inhibiton of the peak current by **2**. Solid curve represents fit to the Hill equation. Data are represented as mean ± SD (n = 3)

As mentioned above, isoprenyl substituents appear to be important for anchoring some natural products to the low voltage-gated Ca^2+^ channel (LVGCC) [19]. The inhibitors of Ca_v_3.1 LVGCC are promising agents for drug development against a number of neurological disorders [20, 21]. In this study, compound **2** was also tested to exhibit strong inhibitory activity on Ca_v_3.1. In HEK 293 cells expressing human Ca_v_3.1, 10 *μ*M **2** notably antagonized currents that elicited by depolarizing voltage steps (− 80 to + 60 mV) (Fig. 4A). It took approximate 120 s to arrive the steady-state inhibition after the perfusion of **2** and this effect was hard to reverse upon washout (Fig. 4B), suggesting that the binding sites of **2** may be buried deeply inside the channel. Dose–response studies showed that **2** inhibited Ca_v_3.1 peak currents with an IC_50_ value of 3.5 *μ*M (Fig. 4C and Table S1). While, the Hill coefficient of **2** is 0.9, indicating a negative cooperativity. Mibefradil, the positive control, indicates a stronger activity than **2**, with an IC_50_ value of 1.12 *μ*M (Fig. 4D and Table S1). Moreover, compounds **1**, **3** and **4** showed negligible effects on Ca_v_3.1 at the higher concentration of 30 *μ*M (Fig. S1).

In conclusion, saldigones A–C (**1, 3, 4**), three new isoprenylated flavonoid constituents featured with diverse flavanone, pterocarpan, and isoflavanone architectures, were characterized from *S. digitanoides,* along with a known isoprenylated flavonone (**2**). To our knowledge, it’s the first report of isoprenylated flavonoid type metabolites from the *Salvia* plants despite many flavonoids have been isolated from this genus [3–8]. In detail, compounds **1** and **2** are isoprenylated flavonoids with methylation and isoprenylation substitutions, while **3** and **4** are two isoprenylated flavonoids with different pterocarpan and isoflavanone skeletons, respectively. In addition, compound **2** was proved to be a potent inhibitor of Ca_v_3.1 LVGCC, indicating that isoprenylated flavonoids are deserved further in-depth exploration in genus *Salvia*. Totally, these findings could enrich diversities of secondary metabolites of the genus *Salvia*, and increased universality of biological activities and properties.

## Experimental

### General Experimental Procedures

Silica gel (100–200 and 200–300 mesh, Qingdao Marine Chemical Co., Ltd., Qingdao, China), MCI gel (75–150 *μ*m, Mitsubishi Chemical Corporation, Tokyo, Japan) were used for column chromatography. Fractions were monitored by TLC (GF 254, Qingdao Marine Chemical Co., Ltd. Qingdao, China), and spots were visualized by heating silica gel plates immersed in 8% H_2_SO_4_ in ethanol. Semi-preparative HPLC was performed on Waters 2695 HPLC with a COSMOSIL C_18_ -MS-II (4.6ID × 250 mm) packed column. 1D and 2D NMR spectra were recorded on a Bruker DRX-600 spectrometer using TMS as an internal standard. Unless otherwise specified, chemical shifts (*δ*) are expressed in ppm with reference to the solvent signals. ESIMS and HRESIMS data were acquired on Agilent G6200 TOF mass spectrometer. Optical rotations were measured on a Jasco P-1020 polarimeter. UV spectra were recorded on a Shimadzu UV-2401PC spectrometer. IR spectra were recorded on a Bruker FT-IR Tensor-27 infrared spectrophotometer with KBr disks.

### Plant Material

Roots of *S. digitaloides* were collected in Haba, Yunnan Province, People’s Republic of China, in August, 2011. The plant material was identified by Dr. Chun-Lei Xiang, Kunming Institute of Botany, Chinese Academy of Sciences. A voucher specimen was deposited in Kunming Institute of Botany, Chinese Academy of Sciences with identification number 20110788.

### Extraction and Isolation

The air-dried roots of *S. digitaloides* (25.0 kg) were extracted with acetone for three times at room temperature (each 100 L, 3 days). The combined extracts were concentrated, the crude extract (730 g) was subjected to a silica gel column chromatography successively eluted with CHCl_3_, EtOAc and MeOH. The EtOAc fraction (180 g) was chromatographed on a silica gel column, eluted with petroleum ether/EtOAc (from 500:1 to 0:1, v/v), to yield five fractions (Fr. A–E) on the basis of TLC analysis. Fraction C (130.0 g) was separated through MPLC on an MCI gel column (MeOH-H_2_O from 60:40 to 100:0) to obtain four fractions (Fr. C1 – C4). Fraction C1 (20.0 g) was then chromatographed on a silica gel column, eluted with petroleum ether/acetone (from 200:1 to 0:1, v/v), to yield six fractions (Fr. C1-1 – C1-6).

Fraction C1-6 (4.0 g) was subjected to column chromatography over a silica gel column eluted with petroleum ether/CHCl3/EtOAc (9/0.7/0.3) to yield seven fractions (C1-6-1 – C1-6-7). Fraction C1-6–2 (900 mg) was chromatographed on Sephadex LH-20 (acetone) and further purified by semi-preparative HPLC (MeCN – H_3_O^+^, 72:28) to afford compounds **1** (2.9 mg, *t*_*R*_ = 11.0 min), **2** (2.7 mg, *t*_*R*_ = 12.9 min), **3** (7.5 mg, *t*_*R*_ = 16.1 min) and **4** (2.2 mg, *t*_*R*_ = 17.3 min).

#### Saldigone A (**1**)

Colorless gum; [*α*]_D_^22^ − 28.3 (*c* 0.08, MeOH); UV (MeOH) λ_max_ (log *ε*) 345 (3.63), 297 (4.27), 195 (5.01), 328 (3.57), 254 (3.55) nm; CD (MeOH; *c* 1.19 × 10^–4^): Δ*ε* 350 (+ 1.74), 293 (− 14.15), 252 (+ 0.47), 242 (− 1.77), 227 (+ 3.57), 215 (− 1.74), 205 (+ 11.52) nm; IR (KBr) *v*_max_ 3411, 2922, 1626, 1607, 1374, 1186, 845, 554 cm^−1^; ^1^H and ^13^C NMR data, see Table 1; ESIMS *m/z* 451 [M – H]^−^; HRESIMS *m/z* 451.2128 [M – H]^−^ (calcd for C_27_H_31_O_6_, 451.2126).

#### Saldigone B (**3**)

White powder; [*α*]_D_^20^ − 187.3 (*c* 0.09, MeOH); UV (MeOH) λ_max_ (log *ε*) 286 (4.06), 208 (5.06), 257 (3.56), 203 (5.04) nm; CD (MeOH; *c* 1.1 × 10^–4^): Δ*ε* 287 (+ 8.35), 236 (− 25.07), 229 (− 22.58), 215 (− 49.89), 203 (+ 19.75) nm; IR (KBr) *v*_max_ 3427, 2924, 1608, 1496, 1354, 1264, 1142, 1124, 1099, 959, 839, 445 cm^−1^; ^1^H and ^13^C NMR data, see Table 1; ESIMS *m/z* 421 [M – H]^−^; HRESIMS *m/z* 421.2021 [M – H]^−^ (calcd for C_26_H_29_O_5_, 421.2020).

#### Saldigone C (**4**)

Pale yellow gum; [*α*]_D_^20^ + 10.9 (*c* 0.08, MeOH); UV (MeOH) λ_max_ (log *ε*) 194 (4.79), 196 (4.81), 254 (3.56), 296 (4,08), 420 (2.35) nm; CD (MeOH; *c* 2.2 × 10^–4^): Δ*ε* 367 (− 0.48), 311 (+ 4.24), 285 (− 3.29), 215 (+ 6.69), 199 (− 10.45) nm; IR (KBr) *v*_max_ 3429, 2924, 1618, 1468, 1178, 989, 567, 486 cm^−1^; ^1^H and ^13^C NMR data, see Table 1; ESIMS *m/z* 437 [M – H]^−^; HRESIMS *m/z* 437.1968 [M – H]^−^ (calcd for C_26_H_29_O_6_, 437.1970).

#### Cell Preparation and Expression

Human embryonic kidney (HEK) 293 cells were grown in DMEM (HyClone) plus 10% newborn calf serum (Gibco) and penicillin (100 U/ml)/streptomycin (0.1 mg/ml) (Biological Industries). HEK 293 cells were transiently transfected with pCDNA3.1-T type and pCDNA3.1-EGFP plasmids together using LipoD293™ (SignaGen Laboratories) and used in 48 h.

## Supplementary Information

Below is the link to the electronic supplementary material.

Supplementary file1 (PDF 1562 kb)
