# Correction to: Isoprenylated Flavonoids as Ca_v_3.1 Low Voltage-Gated Ca^2+^ Channel Inhibitors from *Salvia digitaloides*

**DOI:** 10.1007/s13659-021-00308-x

**Published:** 2021-05-12

**Authors:** Jian-Jun Zhao, Song-Yu Li, Fan Xia, Ya-Li Hu, Yin Nian, Gang Xu

**Affiliations:** 1 State Key Laboratory of Phytochemistry and Plant Resources in West China, Kunming Institute of Botany, Chinese Academy of Sciences, and Yunnan Key Laboratory of Natural Medicinal Chemistry, Kunming, 650201 People’s Republic of China; 2 University of Chinese Academy of Sciences, Beijing, 100049 People’s Republic of China

## Correction to: Natural Products and Bioprospecting 10.1007/s13659-021-00307-y

In the original publication, the authors contributing equally to this paper was not presented clear.

The corrected text is given below:

Jian-Jun Zhao and Song-Yu Li have contributed equally to this work.

