# Supplementary material for: Isoprenylated Flavonoids as Cav3.1 Low Voltage-Gated Ca2+ Channel Inhibitors from Salvia digitaloides
Source: Nat Prod Bioprospect. 2021 Apr 24;11(6):671–8. doi: 10.1007/s13659-021-00307-y (PMC8599534; doi:10.1007/s13659-021-00307-y)
Supplement: Supplementary file 1 — Supplementary file1 (PDF 1562 kb) [file 13659_2021_307_MOESM1_ESM.pdf]

Isoprenylated Flavonoids as $Ca_v3.1$ Low Volage-Gated Ca^{2+} Channel Inhibitors from *Salvia digitaloides*

Jian-Jun Zhao, Song-Yu Li, Fan Xia, Ya-Li Hu, Yin Nian, Gang Xu

Supporting Information

Table of contents

- SI-1. Biological assay (Page S2–S4)
- SI-2. CD spectra of compounds **1**, **3**, **4** (Page S5–S6)
- SI-3. The original NMR and MS spectra of compounds **1**, **3**, **4** (Page S7–S18)

SI-1. Biological assay:

Electrophysiology. All experiments were performed at room temperature (~22 °C). Pipettes were fabricated from borosilicate glass (World Precision Instru-ments) using a micropipette puller (P-1000, Sutter Instrument), and were fire-polished to resistances of 2~4 M for whole-cell recording. Whole-cell cur-rents were elicited by 150 ms depolarization to −40 mV at 4 s intervals from a holding potential (HP) of −100 mV. Currents were amplified by Axopatch 200B and digitized by Digidata 1440A (Molecular Devices). Currents were low-pass filtered at 2 kHz and sampled at 10 kHz. pCLAMP 10 software (Molecular Devices) was used for data acquisition and analysis. The extracellular solutions contained (in mM) 142 CsCl, 1 $MgCl_2$, 2 $CaCl_2$, 10 Glucose and 10 HEPES (pH 7.4 adjusted with CsOH). The intracellular solutions contained (in mM) 127 Cs-methanesulphonate, 2$MgCl_2$, 2Na_2ATP, 10 HEPES and 11 EGTA (pH 7.4 adjusted with CsOH).

Data analysis and statistics. Data fitting and statistical analyses were per-formed using GraphPad Prism 7 (GraphPad Software Company., San Diego, CA, USA). IC_{50} value and Hill coefficient were determined by fitting the data points to a Hill equation with the form of $Y = I_{Min} + (I_{Max} - I_{Min})/[1+10(LogIC_{50}-C)\times Hillslope]$. Where IC_{50} is the concentration at which half-maximal currents were inhibited, C is the concentration of compounds, I_{Min} is the minimum inhibition ratio, I_{Max} is the maximum inhibition ratio, and Hillslope is the Hill coefficient. All the data were presented as mean ± SD.

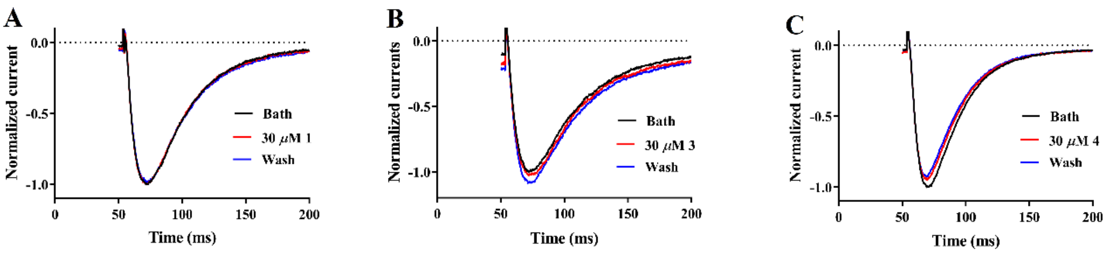

Fig. S1. Compounds **1**, **3**, and **4** are inactive on Cav3.1. A-C: Representative Cav3.1 peak current traces that elicited by 150 ms depolarization to −40 mV at 4 s intervals from a holding potential (HP) of −100 mV in the absence (Bath) and presence of 30 μM **1**, **3**, and **4**.

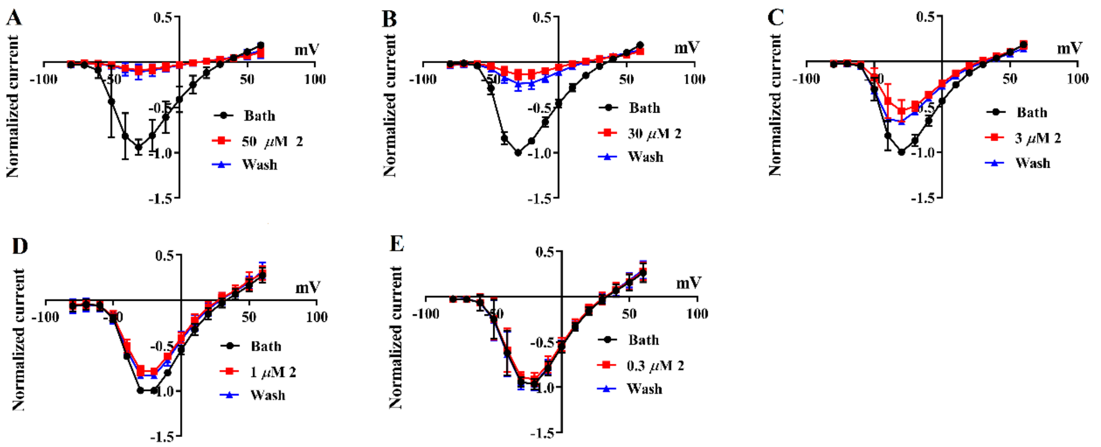

Fig. S2. Normalized current-voltage (I-V) curves of $Ca_V3.1$ in the absence or presence of various concentrations of compound **2** (A-E). $Ca_V3.1$ Currents were evoked from a HP of -100 mV by 150 ms depolarization from -80 mV to + 60 mV in 10 mV increasement at 4 s intervals. All the data were represented as mean ± SD (n=3).

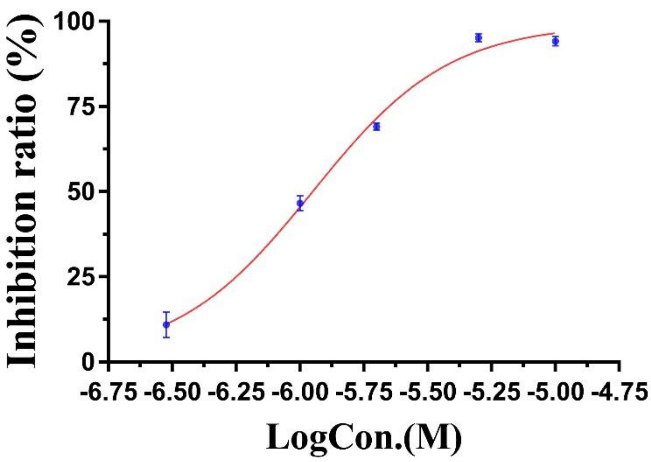

Fig. S3. Dose-response relationships of mibefradil for $Ca_v3.1$. The solid curve represents a fit to the Hill equation with IC_{50} value of 1.12 μM and a Hill coefficient of 1.6. All the data were represented as mean ± SD (n=3).

Table S1. Dose-dependent effects of compound **2** and mibefradil on peak currents of $Ca_v3.1$.

	concentration (μM)	Inhibitory ratio (%)			mean ± SD
		Exp. 1	Exp. 2	Exp. 3	
**Compound 2**	**0.3**	6.2	5.2	3.6	5.0 ± 1.3 %
	**1**	17.4	23.5	21.1	20.7 ± 3.1 %
	**3**	36.8	40.4	57.0	44.7 ± 10.8 %
	**10**	66.5	61.6	76.0	68.0 ± 7.3 %
	**30**	80.0	87.5	91.3	86.3 ± 5.8 %
	**50**	89.1	96.3	87.5	91.0 ± 4.7 %
**Mibefradil**	**0.3**	7.4	14.9	10.5	10.9 ± 3.8 %
	**1**	44.2	46.4	49.3	46.6 ± 2.6 %
	**2**	68.2	70.2	69.1	69.2 ± 1.0 %
	**5**	95.7	93.9	96.1	95.2 ± 1.2 %
	**10**	95.5	92.8	94.2	94.2 ± 1.4 %

SI-2. CD spectra of compounds 1, 3, 4:

Compound 1:

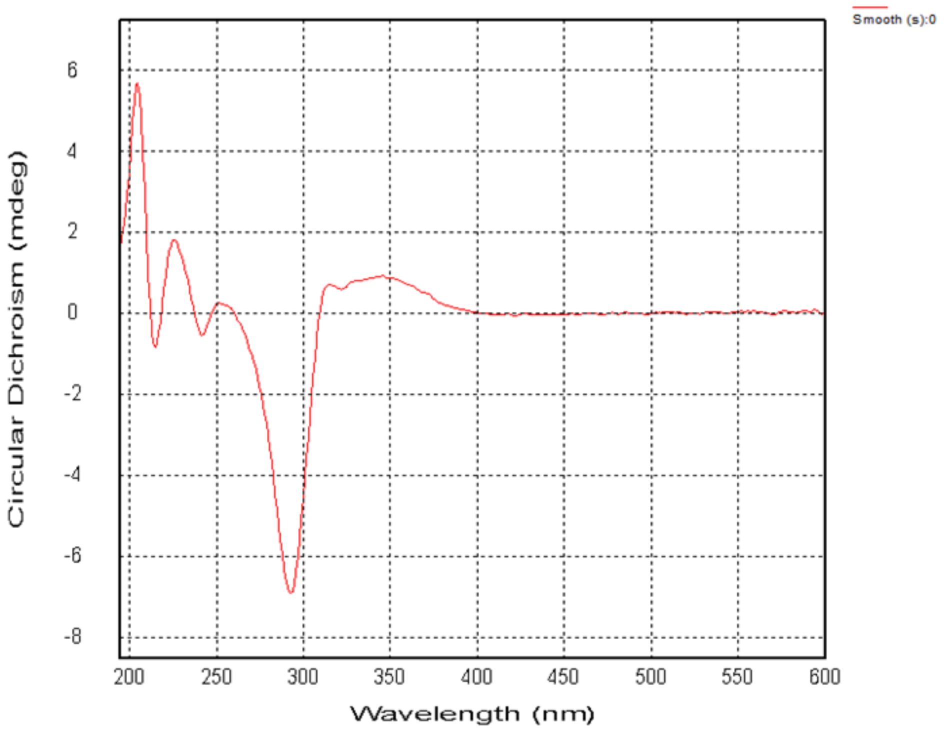

Compound 3:

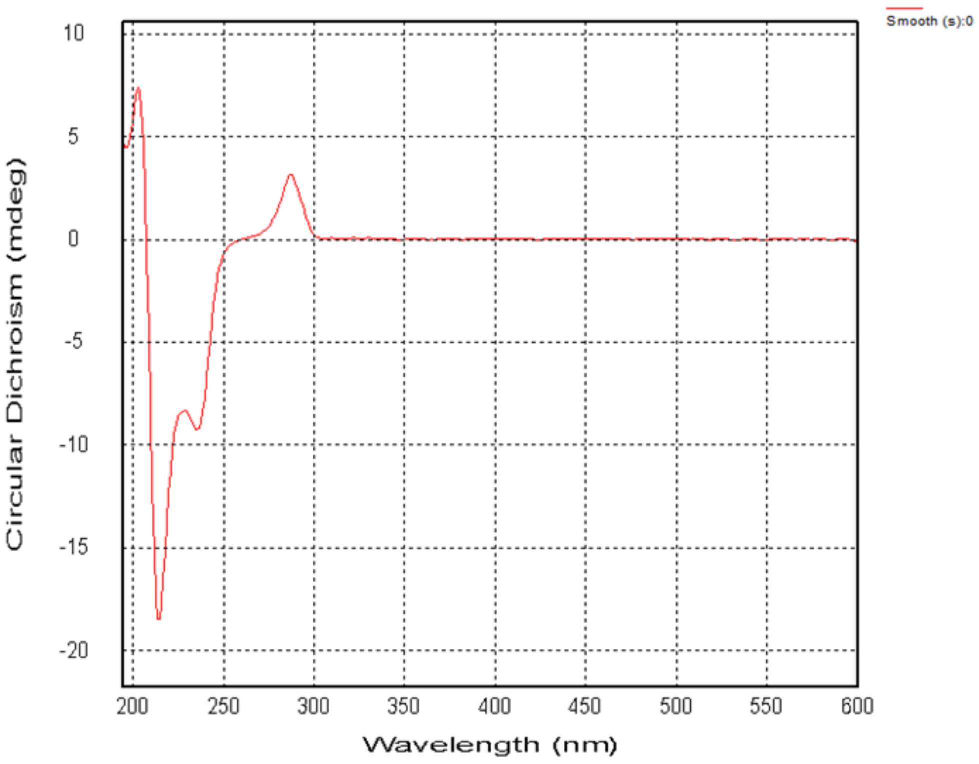

Compound 4:

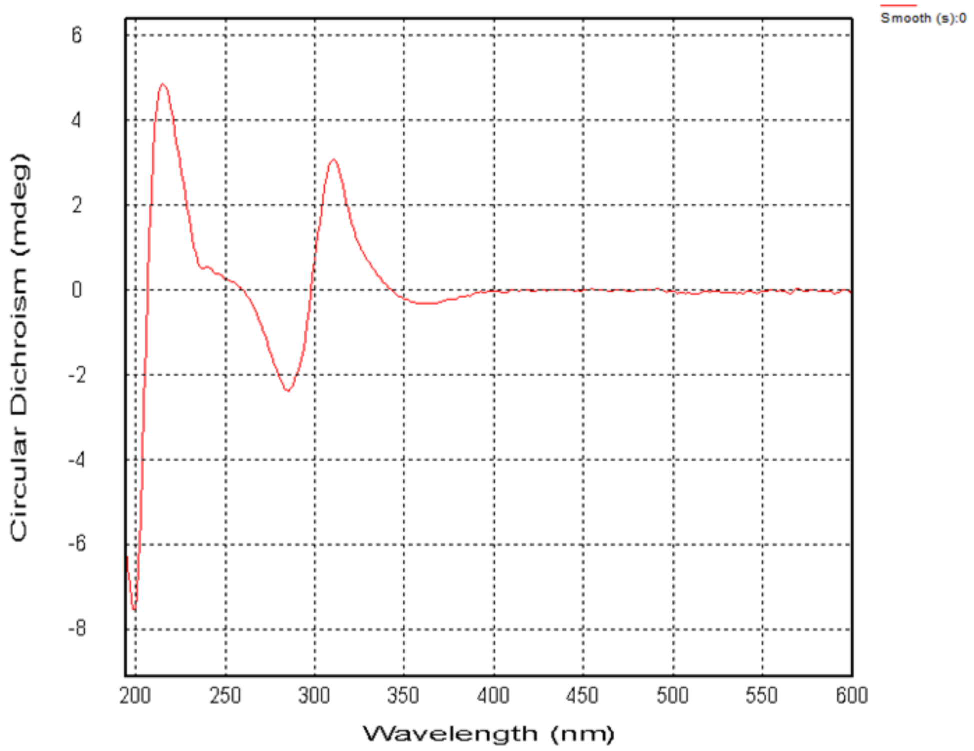
Smooth (s):0
Circular Dichroism (mdeg)
6
4
2
0
-2
-4
-6
-8
200
250
300
350
400
450
500
550
600
Wavelength (nm)

SI-3. The original NMR an d MS spectra of compounds 1, 3, 4

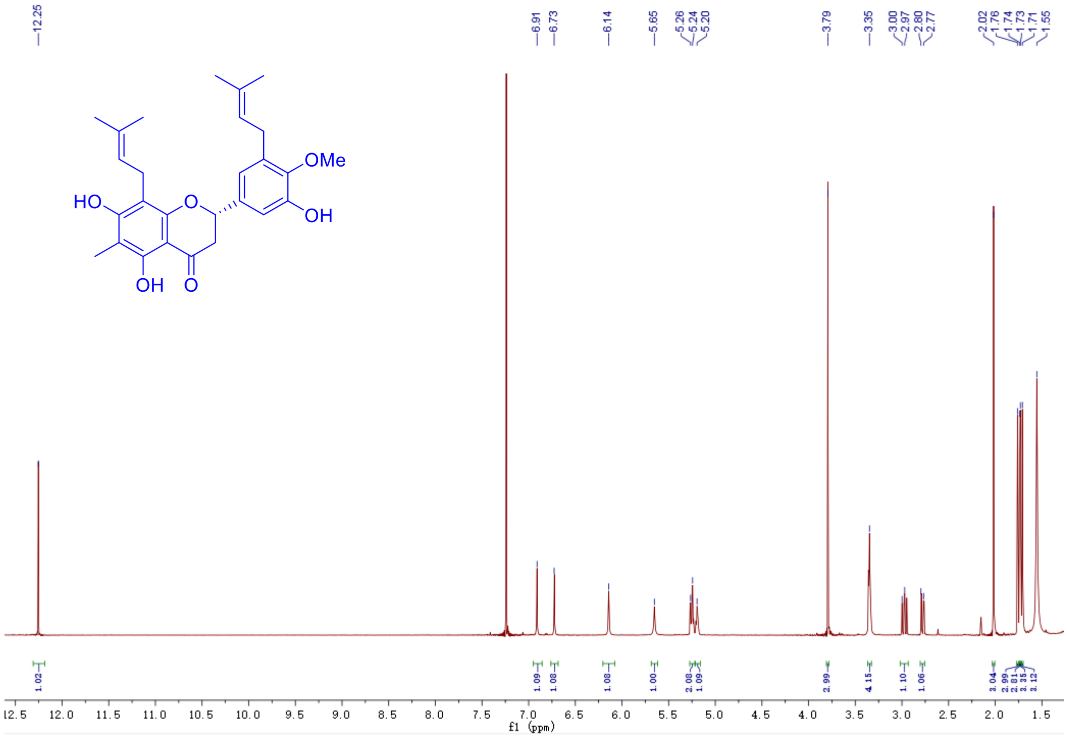

Fig. S4. ^{1}H (in $CDCl_3$) spectrum of saldigone A (**1**).

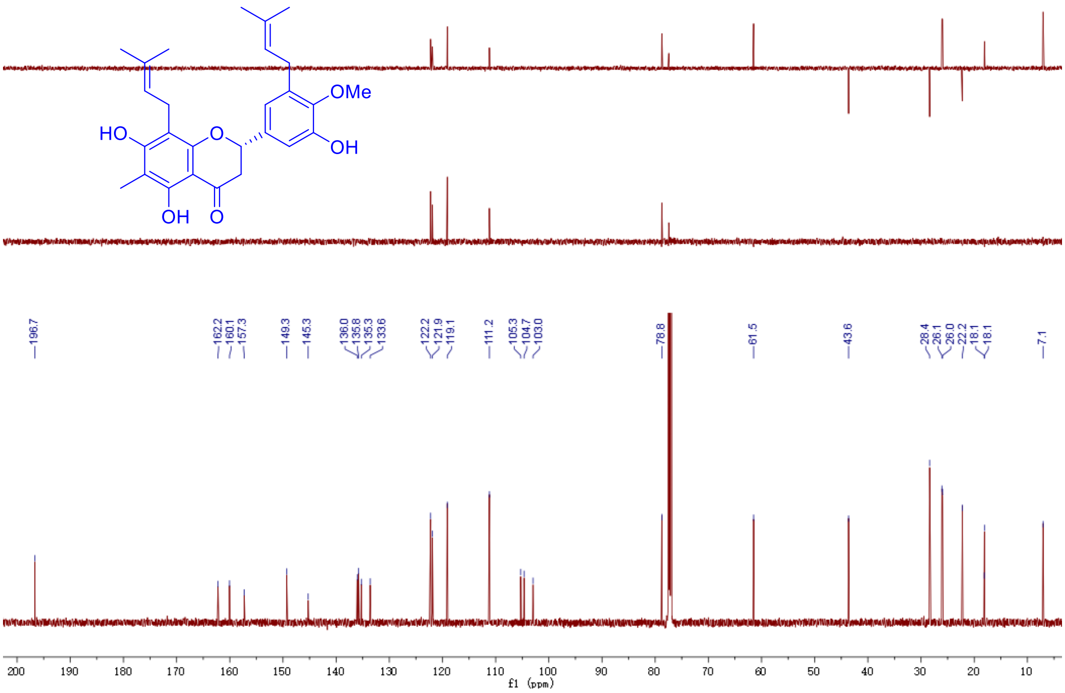

Fig. S5. ^{13}C and DEPT (in $CDCl_3$) spectrum of saldigone A (**1**).

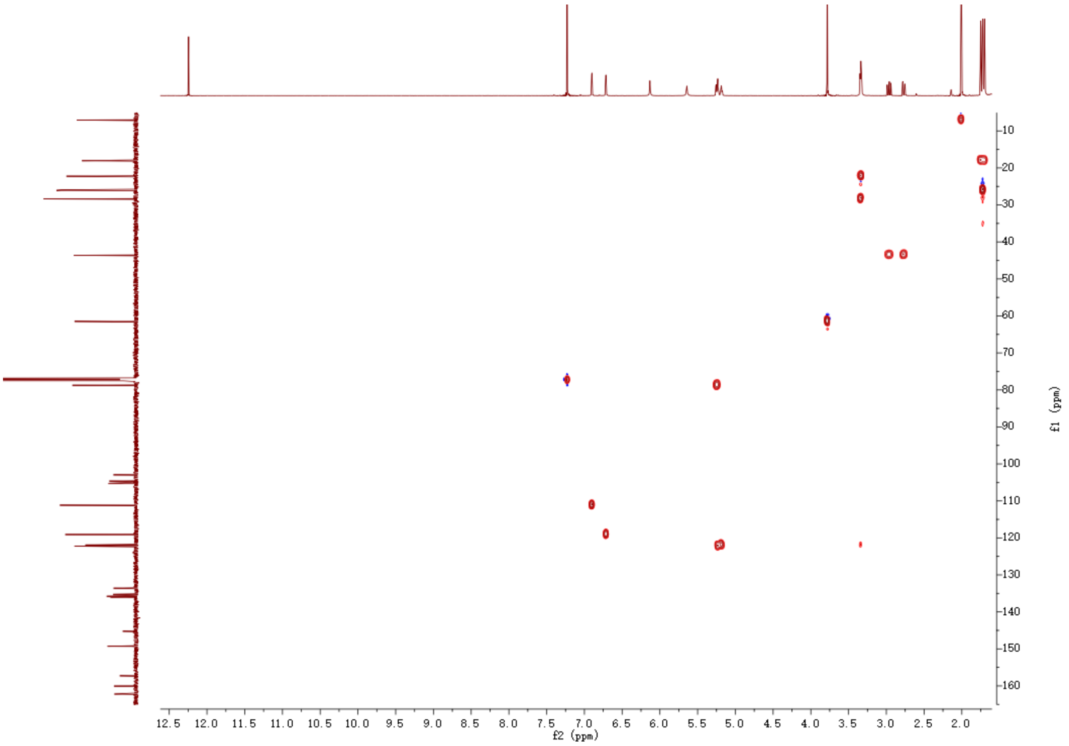

Fig. S6. HSQC spectrum of saldigoneA (**1**).

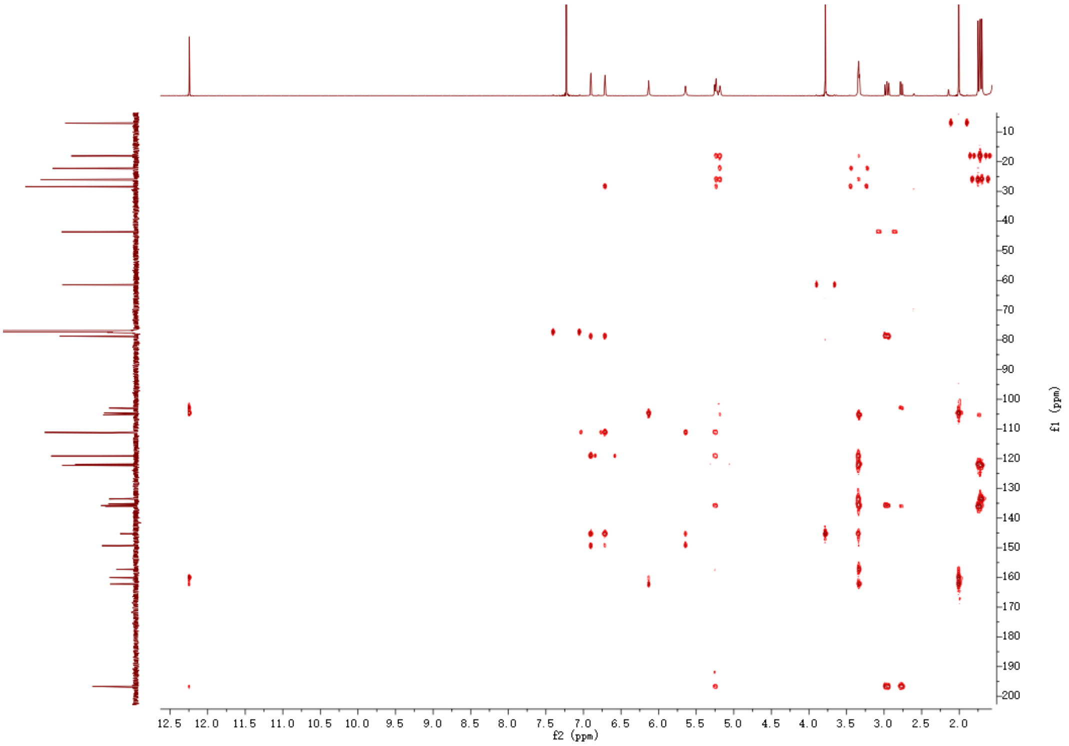

Fig. S7. HMBC spectrum of saldigone A (**1**).

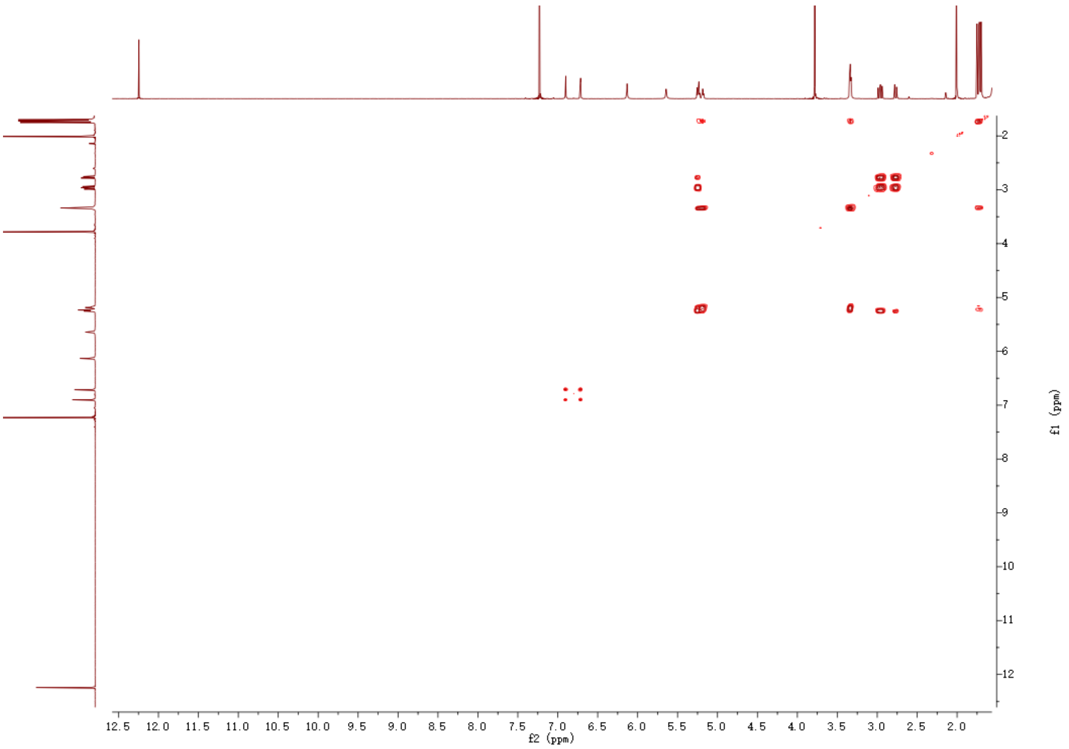

Fig. S8. ^{1}H–^{1}H COSY spectrum of saldigoneA (**1**).

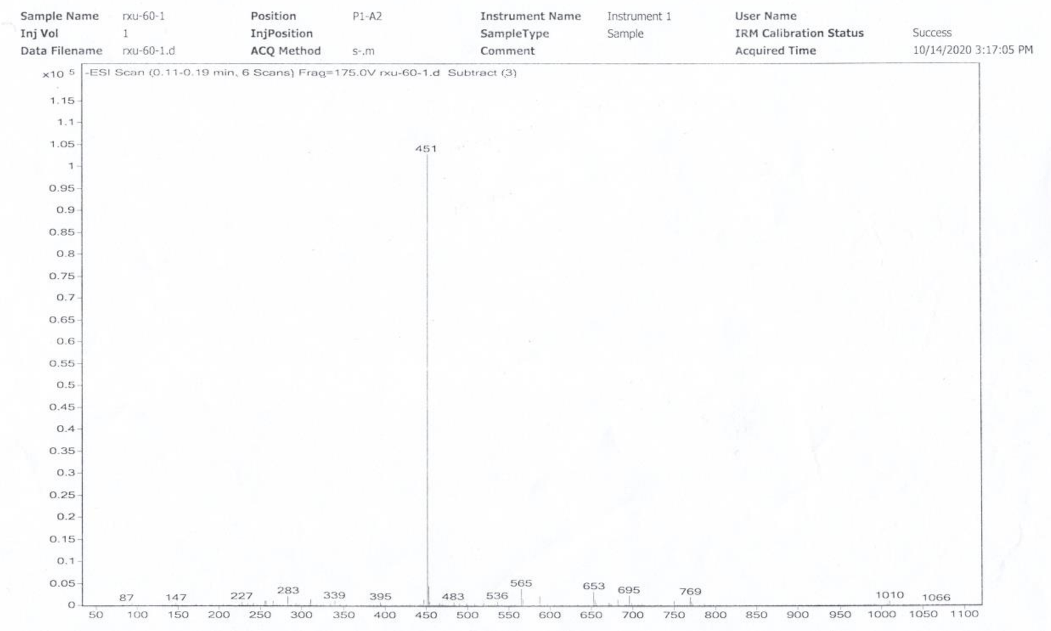

Fig. S9. ESIMS spectrum of saldigone A (**1**).

Qualitative Analysis Report

Data Filename	rxu-60.d	Sample Name	rxu-60
Sample Type	Sample	Position	P1-B2
Instrument Name	Instrument 1	User Name	
Acq Method	s-.m	Acquired Time	10/20/2020 9:41:05 AM
IRM Calibration Status	Success	DA Method	Default.m
Comment			

Sample Group — Info.

Acquisition SW Version: 6200 series TOF/6500 series Q-TOF B.05.01 (B5125.2)

User Spectra

Fragmentor Voltage	Collision Energy	Ionization Mode
175	0	ESI

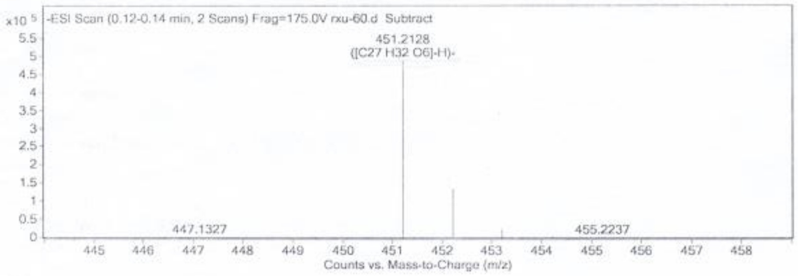

Peak List

m/z	z	Abund	Formula	Ion
112.9857	1	11837.1		
255.2329	1	7639.54		
283.2637	1	3415.44		
451.2128	1	488167.94	C27 H32 O6	(M-H)-
452.2165	1	132730.66	C27 H32 O6	(M-H)-
453.2188	1	23261.66	C27 H32 O6	(M-H)-
454.2216	1	3001.62	C27 H32 O6	(M-H)-
565.2054	1	2741.51		
681.2947	1	4169.35		
955.9711		3587.41		

Formula Calculator Element Limits

Element	Min	Max
C	3	60
H	0	120
O	0	30

Formula Calculator Results

Formula	CalculatedMass	CalculatedMz	Mz	Diff. (mDa)	Diff. (ppm)	DBE
C27 H32 O6	452.2199	451.2126	451.2128	-0.20	-0.44	12.0000

--- End Of Report ---

Fig. S10. HRESIMS spectrum of saldigone A (**1**).

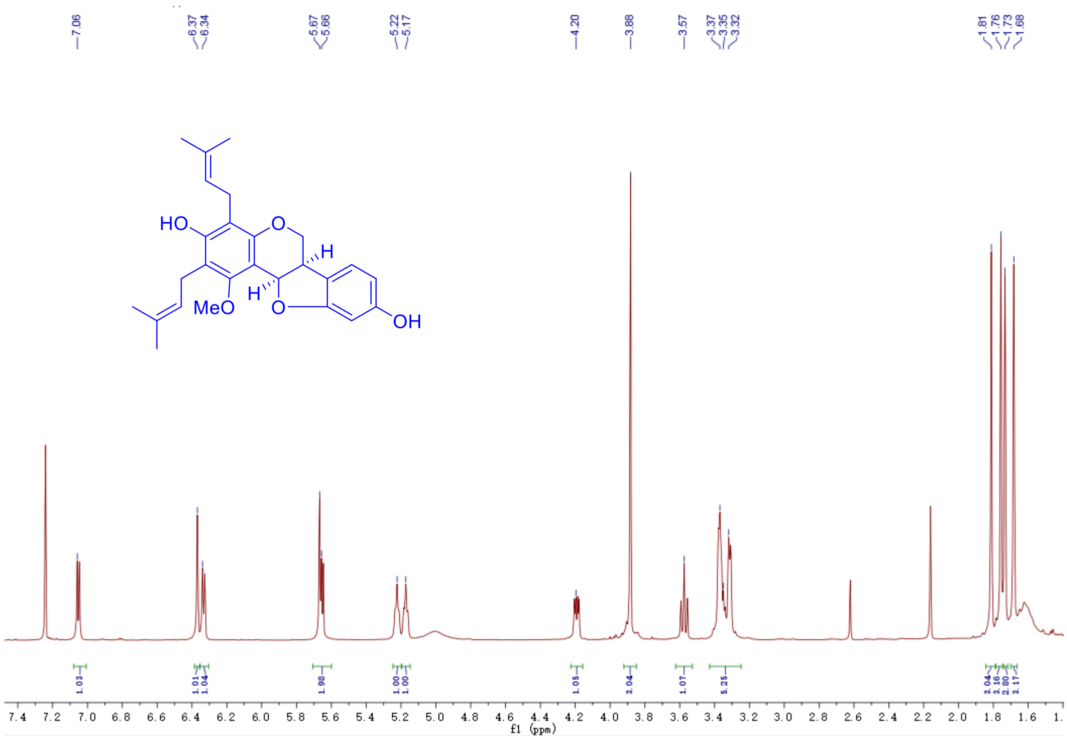

Fig. S11. ^{1}H (in $CDCl_3$) spectrum of saldigone B (**3**).

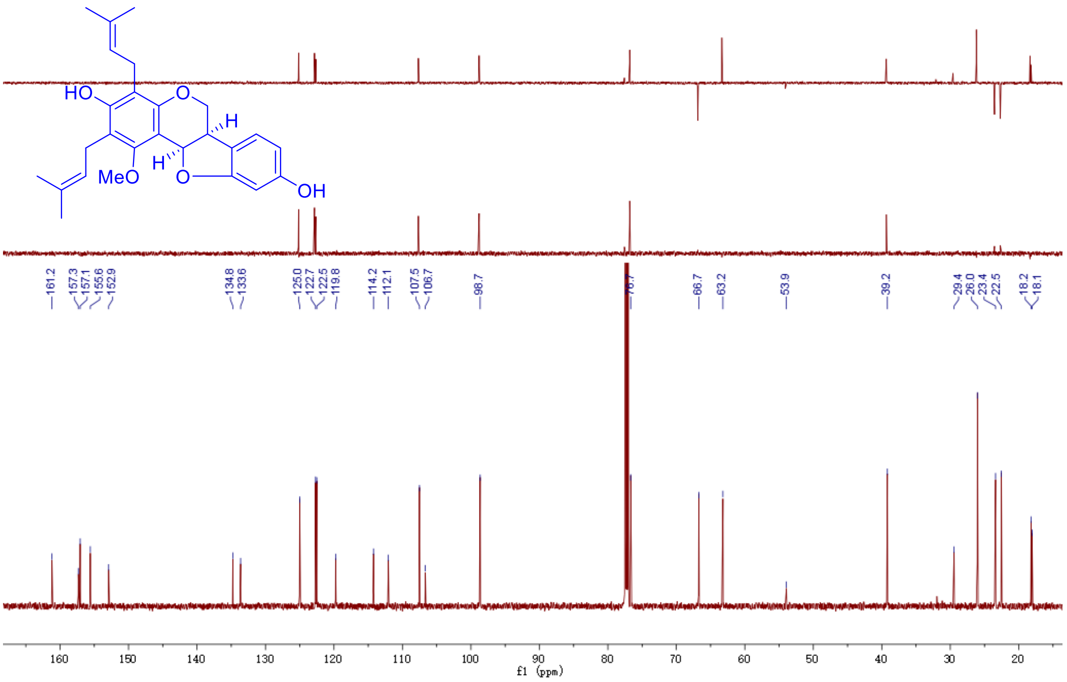

Fig. S12. ^{13}C and DEPT (in $CDCl_3$) spectrum of saldigone B (**3**).

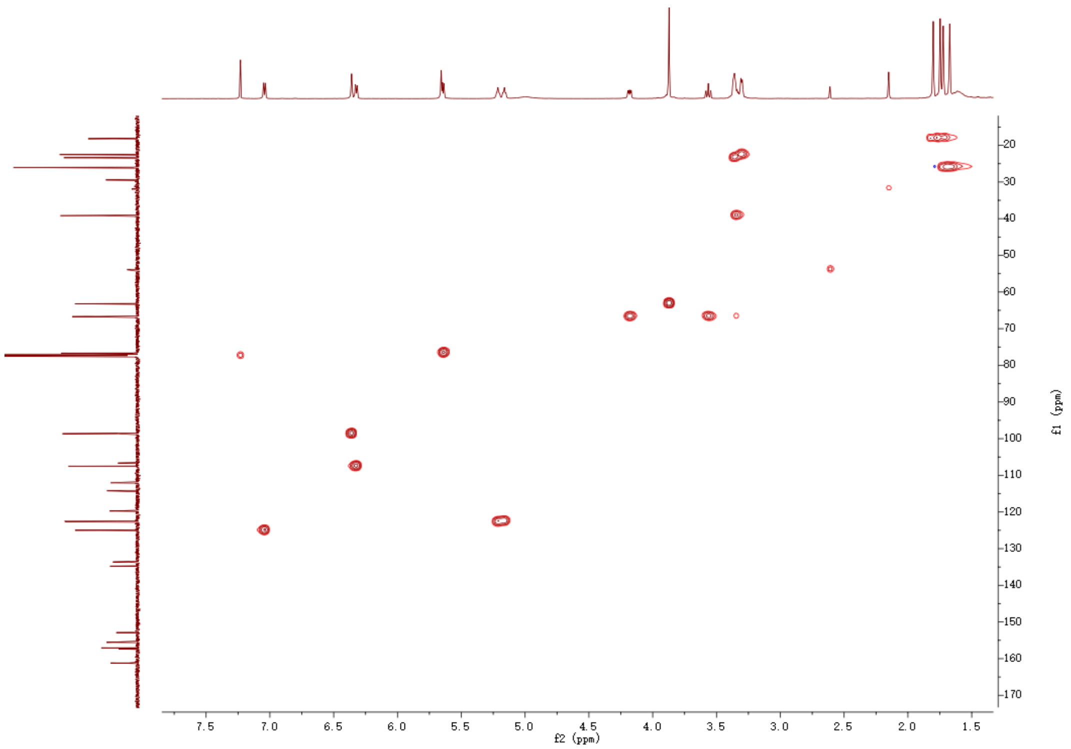

Fig. S13. HSQC spectrum of saldigone B (**3**).

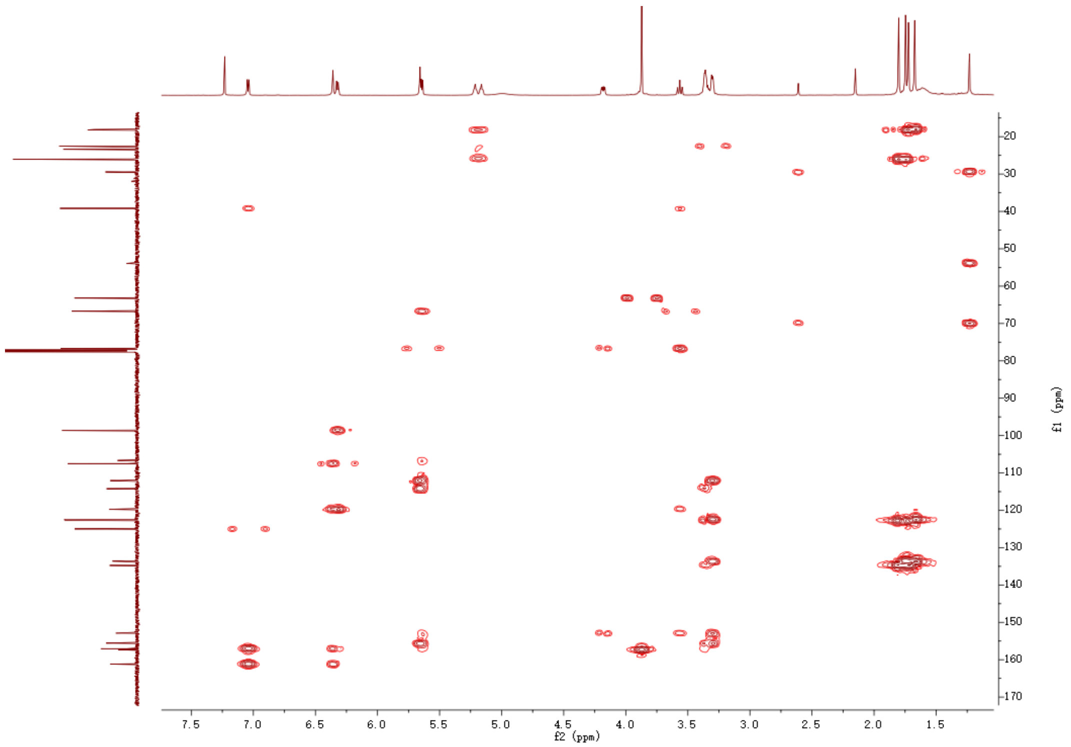

Fig. S14. HMBC spectrum of saldigone B (**3**).

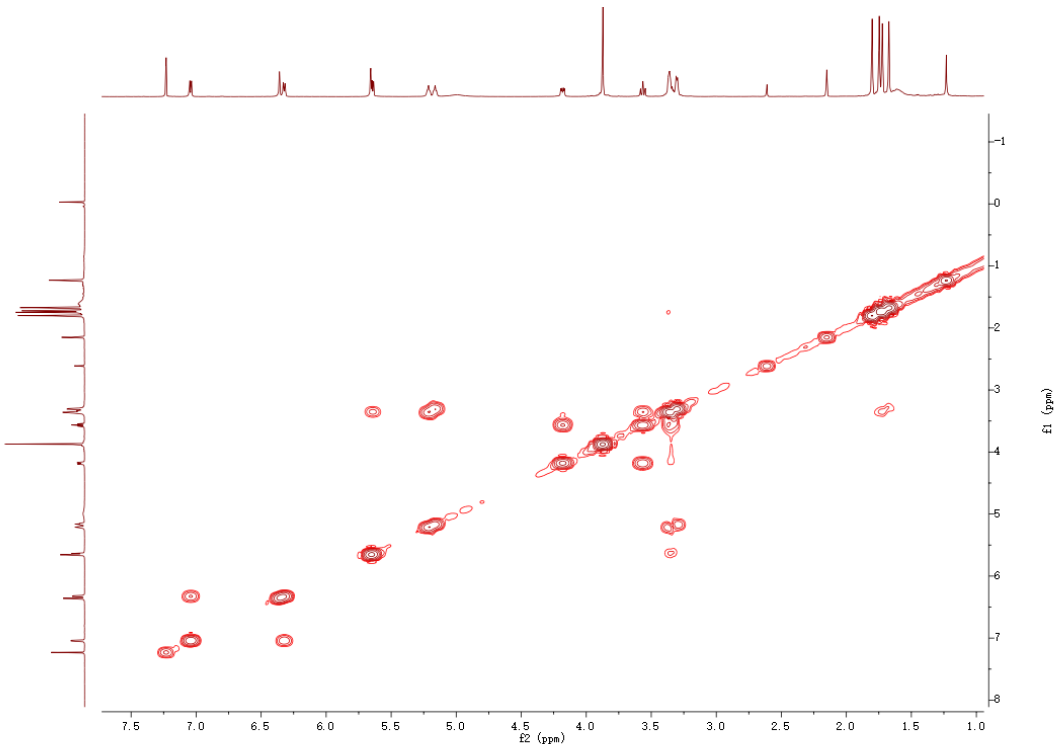

Fig. S15. ^{1}H–^{1}H COSY spectrum of saldigone B (**3**).

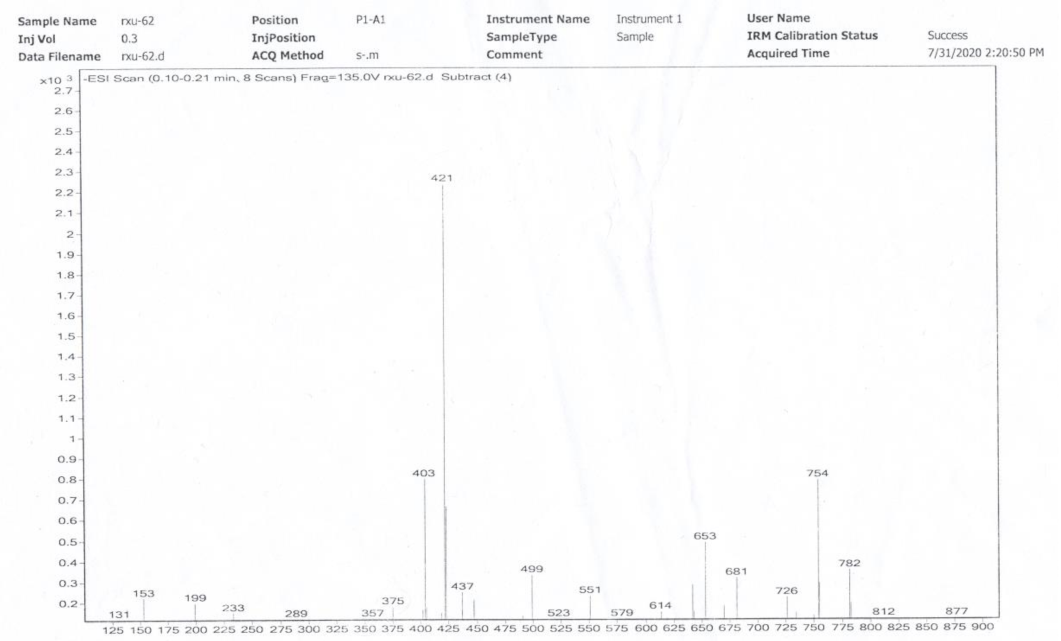

Fig. S16. ESIMS spectrum of saldigone B (**3**).

Qualitative Analysis Report

Data Filename	rxu-62.d	Sample Name	rxu-62
Sample Type	Sample	Position	P1-B4
Instrument Name	Instrument 1	User Name	
Acq Method	s-.m	Acquired Time	10/20/2020 9:43:25 AM
IRM Calibration Status	Success	DA Method	Default.m
Comment			

Sample Group   **Info.**

Acquisition SW Version: 6200 series TOF/6500 series Q-TOF B.05.01 (B5125.2)

User Spectra

Fragmentor Voltage	Collision Energy	Ionization Mode
175	0	ESI

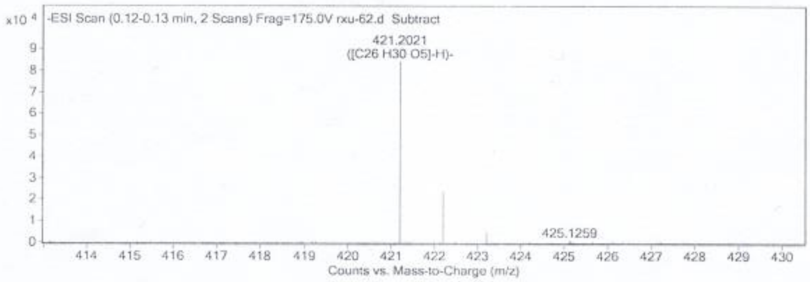

Peak List

m/z	z	Abund	Formula	Ion
112.9856		6186.81		
255.2328	1	16443.24		
283.2638	1	11572.71		
421.2021	1	84258.17	C26 H30 O5	(M-H)-
422.2056	1	24043.63	C26 H30 O5	(M-H)-
423.2074	1	4744.49	C26 H30 O5	(M-H)-
437.1965	1	6300.51		
721.0178	1	4056.65		
982.9904		11766.23		
1033.9881		6238.44		

Formula Calculator Element Limits

Element	Min	Max
C	3	60
H	0	120
O	0	30

Formula Calculator Results

Formula	CalculatedMass	CalculatedMz	Mz	Diff. (mDa)	Diff. (ppm)	DBE
C26 H30 O5	422.2093	421.2020	421.2021	-0.10	-0.24	12.0000

--- End Of Report ---

Agilent Technologies   Page 1 of 1   Printed at: 10:00 AM on: 10/20/2020

Fig. S17. HRESIMS spectrum of saldigone B (**3**).

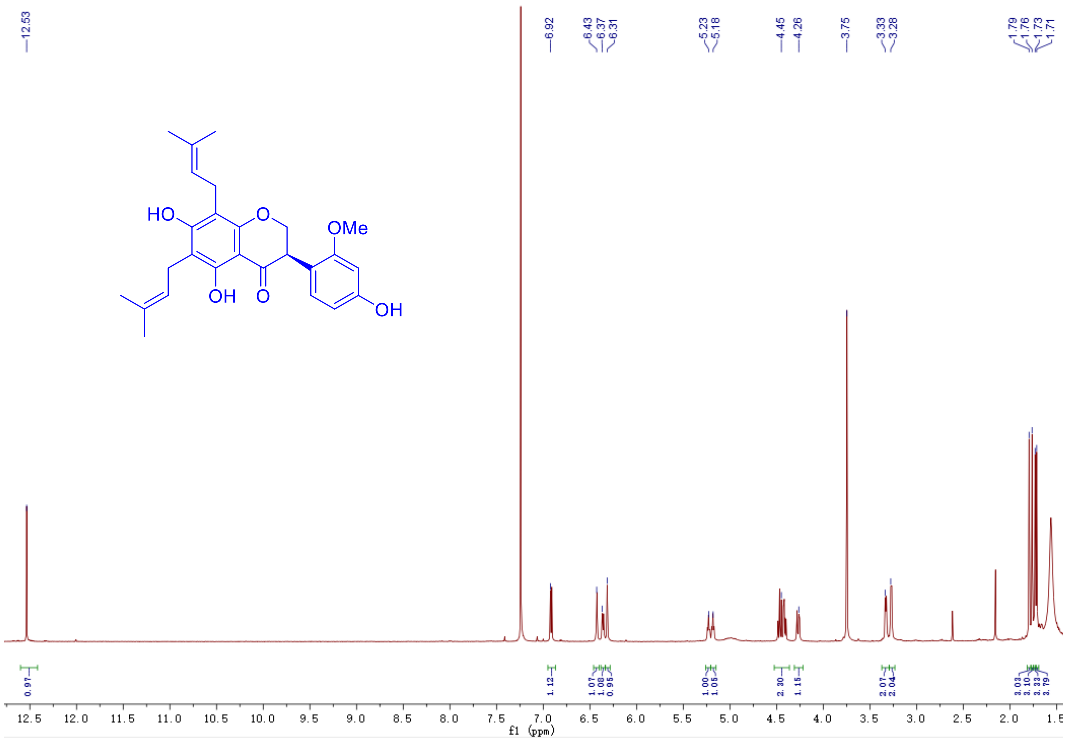

Fig. S18. ^{1}H (in CDCl3) spectrum of saldigone C (**4**).

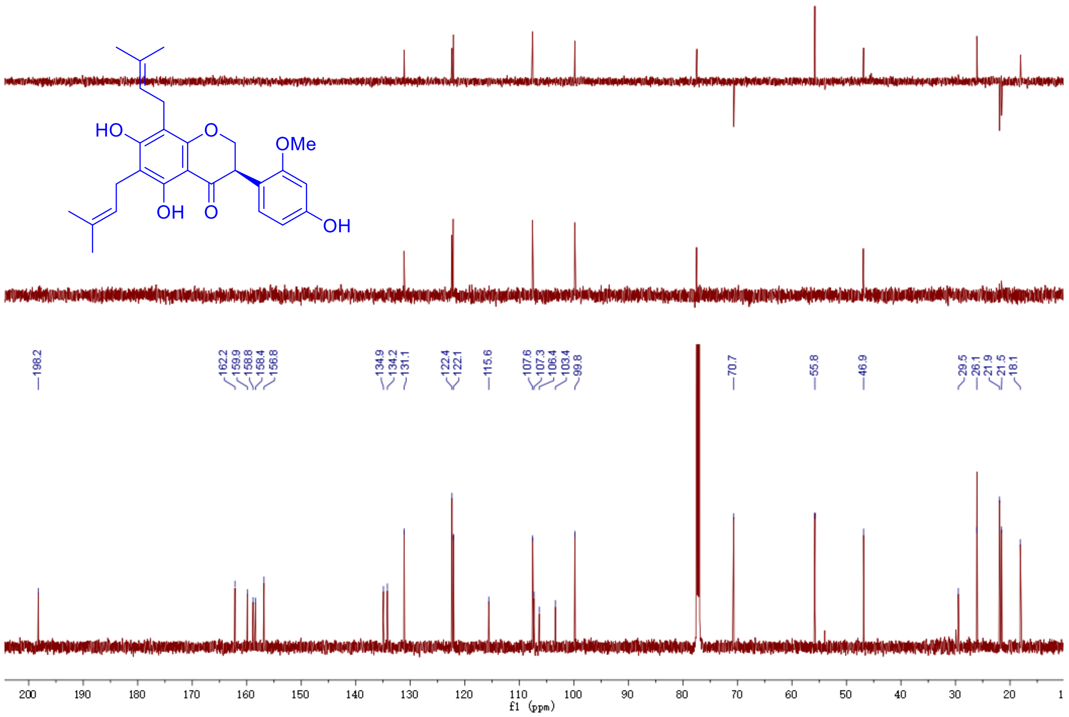

Fig. S19. ^{13}C and DEPT (in CDCl3) spectrum of saldigone C (**4**).

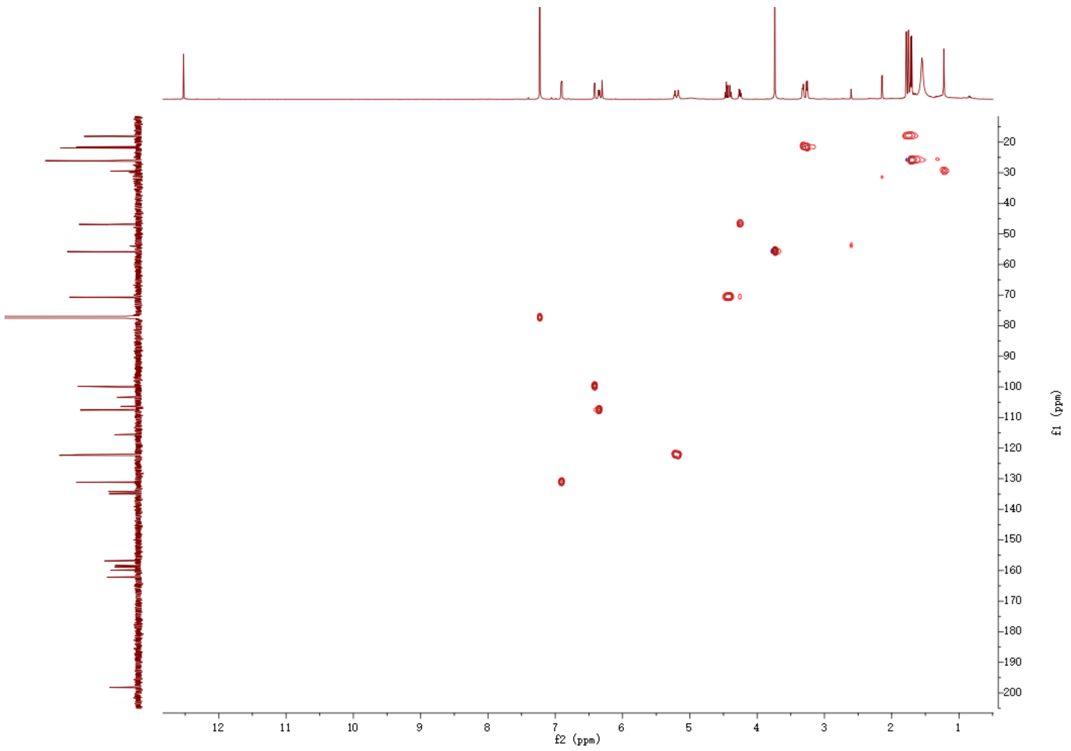

Fig. S20. HSQC spectrum of saldigone C (**4**).

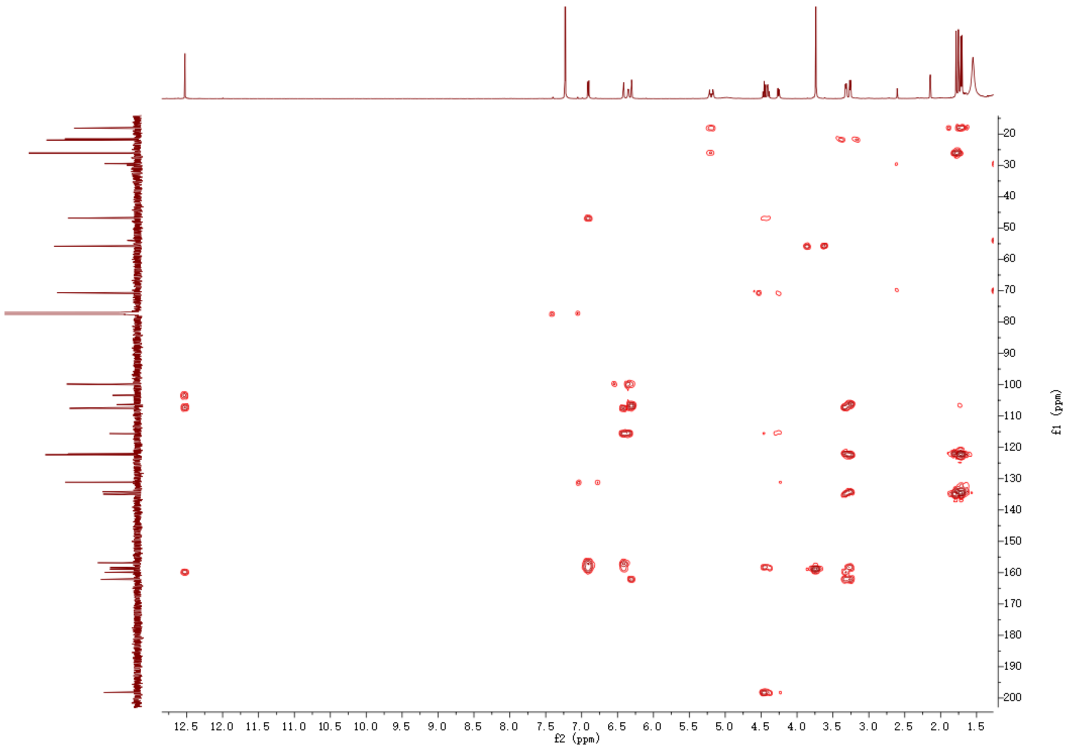

Fig. S21. HMBC spectrum of saldigone C (**4**).

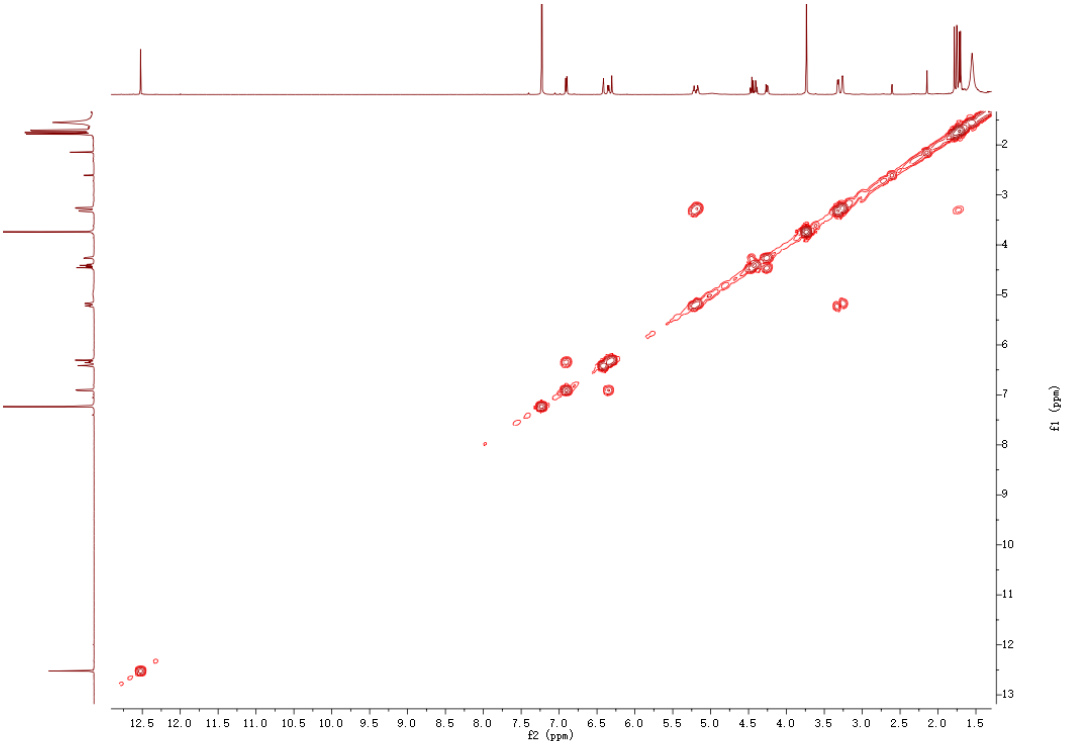

Fig. S22. ^{1}H–^{1}H COSY spectrum of saldigone C (**4**).

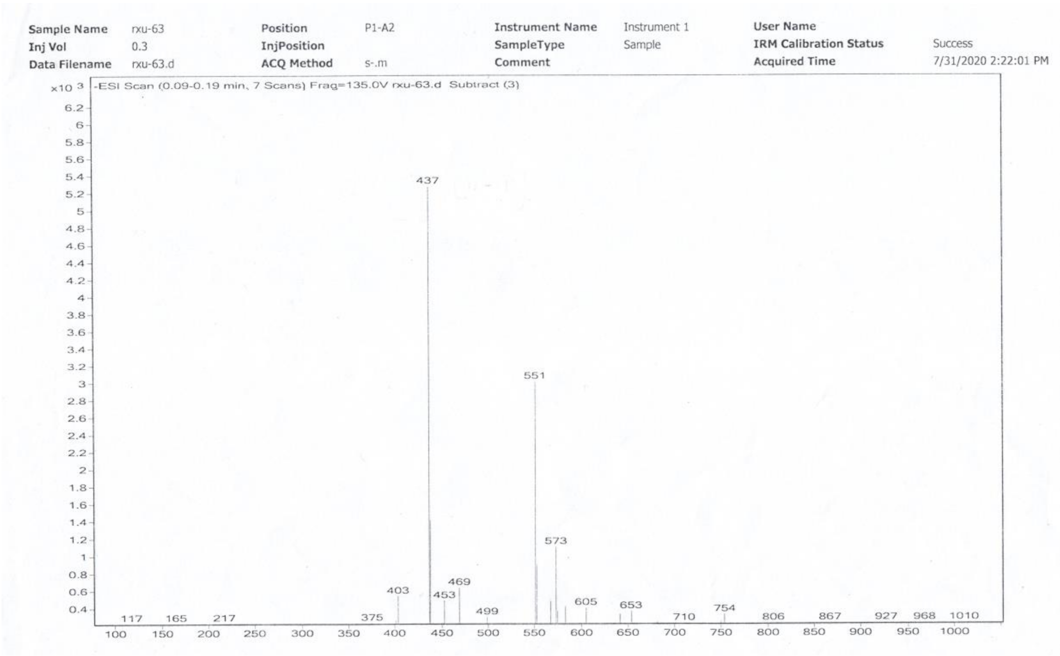

Fig. S23. ESIMS spectrum of saldigone C (**4**).

Qualitative Analysis Report

Data Filename	rxu-63.d	Sample Name	rxu-63
Sample Type	Sample	Position	P1-B5
Instrument Name	Instrument 1	User Name	
Acq Method	s-.m	Acquired Time	10/20/2020 9:44:34 AM
IRM Calibration Status	Success	DA Method	Default.m
Comment			

Sample Group | Info.
Acquisition SW Version | 6200 series TOF/6500 series Q-TOF B.05.01 (B5125.2)

User Spectra

Fragmentor Voltage	Collision Energy	Ionization Mode
175	0	ESI

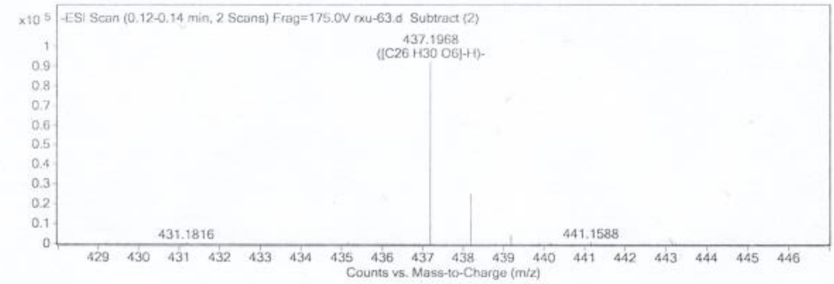

Peak List

m/z	z	Abund	Formula	Ion
207.1389		4013.24		
255.233	1	22184.43		
256.2359	1	4118.75		
283.2641	1	20237.4		
284.2675	1	4531.09		
323.1867	1	8138.88		
437.1968	1	92483.3	C26 H30 O6	(M-H)-
438.2002	1	25582.69	C26 H30 O6	(M-H)-
439.2014	1	4858.08	C26 H30 O6	(M-H)-
453.1904	1	4942.54		

Formula Calculator Element Limits

Element	Min	Max
C	3	60
H	0	120
O	0	30

Formula Calculator Results

Formula	CalculatedMass	CalculatedMz	Mz	Diff. (mDa)	Diff. (ppm)	DBE
C26 H30 O6	438.2042	437.1970	437.1968	0.20	0.46	12.0000

--- End Of Report ---

Agilent Technologies | Page 1 of 1 | Printed at: 10:00 AM on: 10/20/2020

Fig. S24. HRESIMS spectrum of saldigone C (**4**).
